# Effects of Anosognosia on Static and Dynamic Amplitudes of Low-Frequency Fluctuation in Mild Cognitive Impairment

**DOI:** 10.3389/fnagi.2021.705097

**Published:** 2021-09-07

**Authors:** Yanv Fu, Xiao Luo, Qingze Zeng, Kaicheng Li, Tianyi Zhang, Zheyu Li, Xiaopei Xu, Luwei Hong, Yanxing Chen, Minming Zhang, Zhirong Liu

**Affiliations:** ^1^Department of Neurology, The Second Affiliated Hospital of Zhejiang University School of Medicine, Hangzhou, China; ^2^Department of Radiology, The Second Affiliated Hospital of Zhejiang University School of Medicine, Hangzhou, China; ^3^Department of Neurology, Tongde Hospital of Zhejiang Province, Hangzhou, China

**Keywords:** anosognosia, mild cognitive impairment, Alzheimer's disease, amplitudes of low-frequency fluctuation, precuneus, cortical midline structures

## Abstract

**Background:** Anosognosia is a significant symptom in patients with mild cognitive impairment (MCI) while the underlying neurological mechanism behind it is still unclear.

**Methods:** A total of 121 subjects were included and classified into three groups, including 39 normal controls (NCs), 42 individuals with MCI without anosognosia (MCI-NA), and 40 individuals with MCI with anosognosia (MCI-A), based on their everyday cognition (ECog) questionnaire (discrepancy score). Resting-state functional MRIs were acquired from all the subjects, and the static amplitudes of low-frequency fluctuation (sALFF) and dynamic ALFF (dALFF) variance were investigated to evaluate the intrinsic functional network strength and stability, respectively, and both were corrected by age, sex, education, and gray matter volume. Eventually, correlation analyses were conducted to explore the relationship between brain activity changes and cognitive status in all the subjects.

**Results:** No significant difference was found between MCI-A and MCI-NA (*P* > 0.05) in cognitive ability. Regarding intrinsic brain activity, MCI-A had increased sALFF and dALFF variance in the anterior cingulate cortex (ACC) relative to MCI-NA, as well as decreased sALFF and dALFF variance in the precuneus relative to MCI-NA and controls. Moreover, MCI-A had decreased sALFF in the inferior temporal gyrus (ITG) and paracentral lobule (PCL) compared to MCI-NA. Among all the subjects, correlation analyses showed that the sALFF and dALFF variance in the precuneus was related to the Ecog discrepancy score (*r* = 0.232 and 0.235, respectively), immediate story recall (*r* = 0.200 and 0.277, respectively), and delayed story recall (*r* = 0.255 and 0.298, respectively).

**Conclusion:** Alterations of intrinsic brain activation in the ACC and precuneus seem to be associated with the anosognosia symptom in patients with MCI.

## Introduction

A high prevalence of anosognosia symptoms (24.2–71.0%) is reported in patients with Alzheimer's disease (AD) (Mondragon et al., 2019), and the frequency of which is usually associated with the severity of dementia. Previous studies showed that the frequency of anosognosia is around 10% in patients with mild dementia, while in patients with severe dementia, the number rises to 57% (Starkstein et al., 2006; Starkstein, 2014). The term anosognosia was first introduced by Babinski to refer to the phenomenon of denial of hemiplegia (Langer and Levine, 2014). Anosognosia is a syndrome characterized by a lack of awareness of one's illness. Other terms like “unawareness,” “lack of insight,” “impaired self-awareness,” “denial,” and “impaired self-consciousness” have been used to refer to this phenomenon (Markova and Berrios, 2006). Patients with AD having anosognosia are unaware of their cognitive and behavioral deficits (Sunderaraman and Cosentino, 2017; Therriault et al., 2018). Clinically, the onset of anosognosia in AD is significantly related to milder depression and anxiety, severe caregiver burden, and dangerous behaviors (Starkstein, 2014). At the prodromal AD stage, the patients with mild cognitive impairment (MCI) who have anosognosia symptoms are more likely to progress and eventually end up with dementia (Edmonds et al., 2014; Munro et al., 2018; Therriault et al., 2018). This suggests that anosognosia is an independent risk factor of the transition from MCI to AD (Gerretsen et al., 2017).

In previous imaging studies, researchers explored the mechanism behind anosognosia in patients with AD. Specifically, structural MRI studies showed that the severity of anosognosia is associated with greater gray matter atrophy in brain regions like the hippocampus (Tondelli et al., 2018), the anterior cingulate cortex (ACC) (Spalletta et al., 2014), and the superior frontal gyrus (Fujimoto et al., 2017). Meanwhile, a resting-state functional MRI (rs-fMRI) study showed that patients with AD who have anosognosia had reduced intrinsic connectivity between orbitofrontal and posterior cingulate cortex (PCC) (Perrotin et al., 2015). Another task-based fMRI study revealed the association between decreased functional activation of the medial prefrontal cortex (MPFC) and anosognosia in patients with AD during self-appraisal conditions (Zamboni et al., 2013). Notably, the aforementioned brain regions are located in the cortical midline structures (CMS) of the brain, related to the self-referential process (Northoff et al., 2006).

In fMRI approaches, amplitudes of low-frequency fluctuation (ALFF) measures the total power of a given time course within a specific frequency range (Zang et al., 2007). To further elaborate, static ALFF (sALFF) reflects the regional intrinsic functional activity strength by quantifying the average ALFF signal, while the dynamic ALFF (dALFF) mapping reflects the temporal variability of intrinsic brain activity by evaluating the dynamic brain activity using “sliding-window” approaches. The sALFF has been used to investigate the spatial patterns of intrinsic brain activity in patients with MCI and AD (Wang et al., 2011; Liu et al., 2014). Recent fMRI studies have shown that brains are remarkably active even in the absence of overt behavior (Allen et al., 2014). The dALFF could reflect the temporal stability of the intrinsic brain activity and has been used to investigate the time-varying local brain activity in neuropsychiatric disorders, like MCI and depression disorder (Li J. et al., 2019; Yu et al., 2019), providing an opportunity to extract more network information, enhancing our understanding of the properties of brain networks in anosognosia.

Considering that the patients with MCI with anosognosia (MCI-A) had an increased risk of disease progression, we aim to explore the brain intrinsic functional activity in MCI-A by using two fMRI metrics: sALFF and dALFF. We hypothesize that MCI-A will present abnormal brain activity in the CMS regions.

## Materials and Methods

### Participants

Data used in this article were acquired from the AD Neuroimaging Initiative (ADNI) database (www.adni.loni.usc.edu). ADNI is a longitudinal multicenter study since 2004, and now contains ADNI-1, ADNI-GO, ADNI-2, and ADNI-3. By the use of clinical, neuropsychological assessment, gene, biospecimen, and imaging data, it aims to investigate the biomarker of an early detection and progression of AD. For updated information, see www.adni-info.org.

All the subjects in this study signed the written informed consent as they joined the ADNI project. A total of 301 non-demented subjects (characterized as either NC group or MCI) were identified from ADNI databases in January 2020. All subjects underwent structural MRI scans, resting-state functional MRI (rsfMRI) scan, and comprehensive neuropsychological assessments.

**Figure 1:**
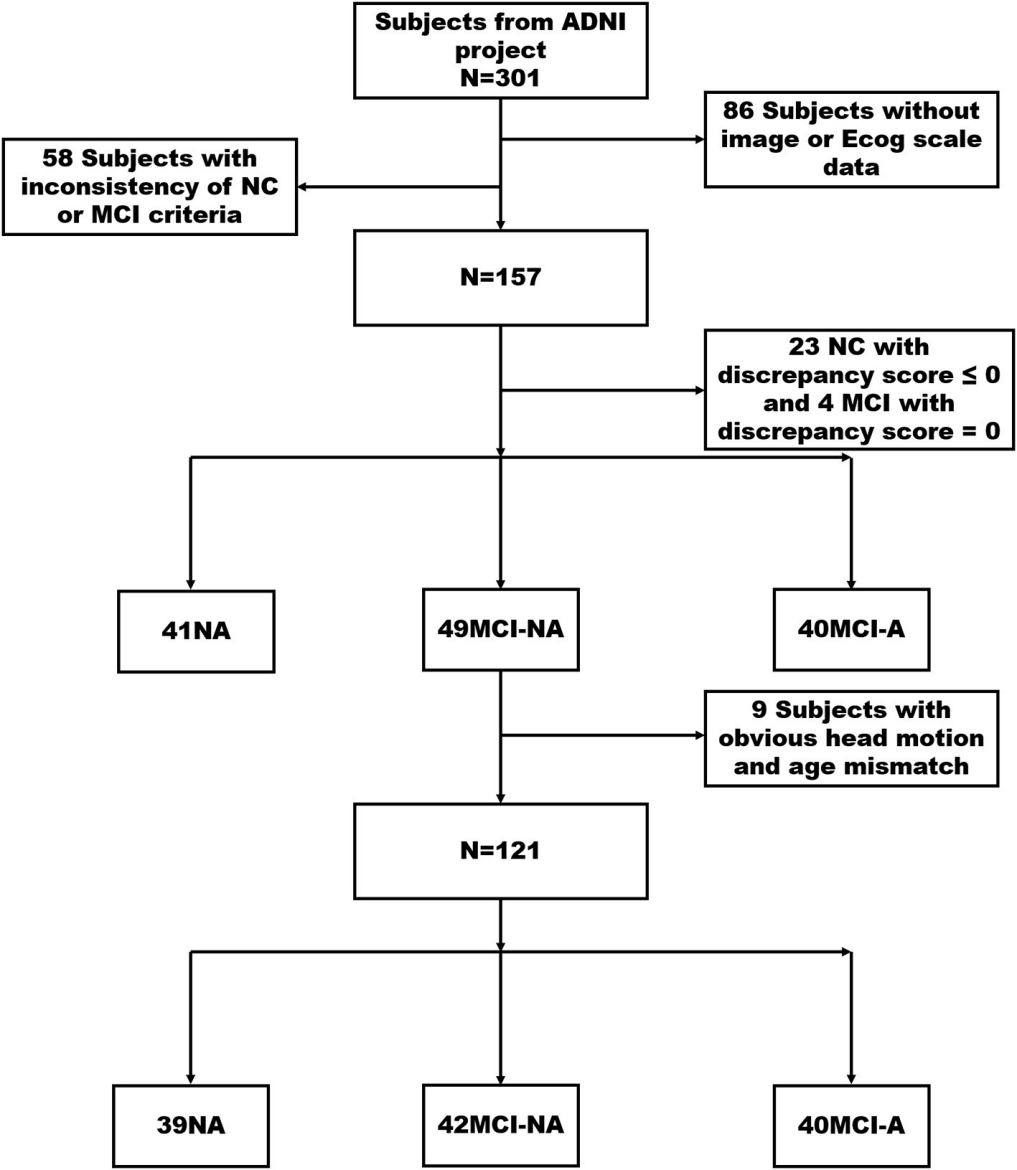
It illustrates the screening processing of NC, MCI-NA, and MCI-A. Finally, 121 participants were divided into three groups consist of 39 NCs (discrepancy score > 0), 42 MCI-NA (discrepancy score > 0), and 40 MCI-A (discrepancy score < 0). MCI, mild cognitive impairment; ECog, Everyday Cognition; NC, normal controls; MCI-A, MCI with anosognosia; MCI-NA, MCI without anosognosia; ADNI, Alzheimer's Disease Neuroimaging Initiative.

Normal controls were defined as: (1) clinical dementia rating (CDR) = 0; (2) mini-mental state exam (MMSE) between 24 and 30 (inclusive); (3) normal Wechsler memory scale logical memory, WMS-LM, delay recall performance (in detail: ≥ 9 for subjects with 16 or more years of education; ≥ 5 for subjects with 8–15 years of education; and ≥ 3 for subjects with 0–7 years of education); (4) absence of significant impairment in cognitive functions or activities of daily living. Subjects with MCI were defined as (1) CDR = 0.5 with a memory box score of at least 0.5; (2) MMSE between 24 and 30 (inclusive); (3) abnormal memory function on the WMS–LM, delay recall (in detail: ≤ 8 for 16 years of education or more years of education, ≤ 4 for 8–15 years of education, and ≤ 2 for 0–7 years of education); (4) general cognition and functional performance sufficiently preserved such that the site physician cannot make a diagnosis of AD at the time of the screening visit. The exclusion criteria include the following: (a) significant medical, neurological, and psychiatric illness; (b) evident head trauma history; (c) use of non-AD-related medication known to influence cerebral function; (d) clinical depression; (e) alcohol or drug abuse. Of the 301 participants, 157 subjects, including 64 NCs and 93 patients with MCI met the inclusion criteria after initial screening (Figure 1).

### Anosognosia Assessment

The severity of anosognosia was evaluated with the discrepancy score between patient and informant global ratings on the ECog questionnaire (Farias et al., 2008), which measures an ability of an individual to perform daily activities compared with 10 years ago. A study partner, such as a spouse or caregiver, who spent a minimum of 10 h per week with the participant, also completed the ECog questionnaire according to the cognitive abilities of the participants. The ECog questionnaire consists of the global cognition scale and 6 neuropsychological subscales, including memory, language, visuospatial, planning, organization, and divided attention. In our study, we choose the memory subscale score calculated by averaging ratings for all 8 items as our data source.

The discrepancy score is obtained by subtracting the study partner report score from the self-report score of the participants. The anosognosia threshold is obtained by using the mean and SD in ECOG discrepancy score from our NC group. In order to better control the variables, we choose the NC group with a discrepancy score > 0 as the selected NC group in our study. Then, we divided these 157 participants into three groups, including 41 NCs, 49 MCI-NA (discrepancy score > 0), and 40 MCI-A (discrepancy score < 0). After excluding the subjects with apparent head motion displacement and age mismatch, 121 non-demented subjects were classified into the following three groups: 39 NCs, 42 MCI-NA, and 40 MCI-A.

### Neuropsychological Assessment

Each subject completed a battery of comprehensive cognitive assessments. Specifically, general cognition was assessed by general mental status (MMSE); memory was accessed by auditory verbal learning test (AVLT), immediate story recall (IST), and the 30-min delayed story recall (DST); attention was accessed by trail-making test part A (TMT-A); executive function was accessed by trail-making test, part B (TMT-B); visuospatial function was accessed by clock-drawing test (CDT), and language abilities were accessed by the semantic verbal fluency (SVF, animal part).

### Neuroimaging Methods

#### Data Acquisition and Pre-processing

The T1-weighted structural images were obtained with the following parameters: voxel size = 1.0 mm × 1.0 mm × 1.2 mm; flip angle = 9°; echo time (TE) = 3.1 ms; inversion time (TI) = 900 ms; repetition time (TR) = 2,300 ms; 170 sagittal slices; within plane FOV = 256 mm × 256 mm. Furthermore, the rs-fMRI images were obtained with an echo-planar imaging sequence using the following parameters: TE = 30 ms; TR = 3,000 ms; the number of slices = 48; slice thickness = 3.3 mm; spatial resolution = 3.31 mm × 3.31 mm × 3.31 mm. All the subjects underwent MRI scans with their eyes open focusing on a cross, and keep at rest calmly according to the ADNI scanning protocol.

Data pre-processing steps were performed using the Data Processing Assistant for Resting-state fMRI (DPARSF, Yan and Zang; http://rfmri.org/dpabi) based on Statistical Parametric Mapping 12 (SPM12; www.fil.ion.ucl.ac.uk/spm). The first ten volumes of the rs-fMRI images were removed due to the signal equilibrium, and the adaptation of the subject to the scanning noise. The remaining 130 images were corrected for both the timing differences between each slice and head motion. Image data with more than 2.5 mm maximum displacement in any of the x, y, or z directions or 2.5° of any angular motion were discarded. T1-weighted images were co-registered to the mean rs-fMRI image and spatially normalized to the Montreal Neurological Institute (MNI) standard space based on rigid-body transformation, then re-sampled into 3 mm × 3 mm × 3 mm cubic voxel. The resulting data were then spatially smoothed with a 6-mm full-width at half-maximum (FWHM) Gaussian kernel to reduce noise and residual differences in gyral anatomy. Then, the removal of linear trends was performed. To control the residual effects of motion and other non-neuronal factors, we concluded covariates, including 24 head motion parameters and signals of white matter and cerebrospinal fluid.

#### sALFF and dALFF Variance Calculation

The sALFF was examined using the DPARSF to reflect the strength of intrinsic brain activity with the following procedures: first, the time series were changed into the frequency domain with a fast Fourier transformation at each voxel; then, across 0.01 to 0.08 Hz, we computed and averaged the square root of the power spectrum, which was taken as the sALFF at the given voxel.

The dALFF was computed using the DynamicBC software (www.restfmri.net/forum/DynamicBC) to reflect the temporal stability of intrinsic brain activity. According to a previous study that window size in the range of 40 to 100s is suitable to capture brain dynamics (Zalesky and Breakspear, 2015; Li K. C. et al., 2019). Thus, we chose 20TR (the 60s) as window size and 1TR as window step. Then, we obtained an ALFF map for each sliding window, as well as the dALFF variance, which reflects the temporal stability of intrinsic brain activity. The dALFF in other window sizes (14TR, 26TR, and 33TR) with 1TR as the window step were also analyzed (Supplementary Table 1 and Supplementary Figures 1, 2). We repeated statistical analysis by using different window sizes (14TR, 20TR, 26TR, and 33TR) and step width (2TR, 3TR) (Supplementary Table 4 and Supplementary Figure 3).

#### Statistical Analysis

Demographics were analyzed with SPSS (version 23.0) using a chi-squared test for categorical data (gender) and ANOVA for continuous data (e.g., age, education). ANCOVA was performed to explore the neuroimaging metric differences (including sALFF and dALFF) between different groups with the gray matter volume and age, sex, education as covariances. We also repeated ALFF analysis using ANCOVA controlling for age, sex, education, gray matter volume, and head motion (Supplementary Table 2). Multiple comparisons correction was performed using the Gaussian random field (GRF) method by setting *P* < 0.01 at the height level and *P* < 0.05 at the cluster level. Then, *post-hoc* analysis was performed to explore the difference between groups by setting the threshold at *P* < 0.05. Furthermore, the partial correlation was conducted to investigate the relationship between neuroimaging metrics (sALFF and dALFF variance in ROIs) and neuropsychological test scores corrected by age, sex, education, and gray matter volume.

## Results

### Demographics

There is no significant difference in age, head motion, and education among the three groups (*P* > 0.05). Regarding neuropsychological scores, MCI-A had lower neuropsychological test scores, including MMSE, IST, DST, AVLT, and SVF, compared to the healthy controls (*P* < 0.05). Moreover, there is no significant difference found between MCI-A and MCI-NA in the neuropsychological scores (*P* > 0.05, Table 1).

**Table 1:** Demographics information and neuropsychological scale of NC, MCI-NA, and MCI-A.

**Demographic**	**NC**	**MCI-NA**	**MCI-A**	**F/χ2**	***P*-value**
**characteristics**	***N* = 39**	***N* = 42**	***N* = 40**		
ECOG	0.64 (0.46)	0.93 (0.57)	−0.78 (0.52)	126.01	0.000
Age	74.9 (6.03)	73.01 (6.84)	73.51 (6.66)	0.90	0.409
Education	16.44 (2.77)	16.45 (2.68)	16.73 (2.47)	0.15	0.860
Gender (F/M)	23/16	20/22	13/27	5.61	0.060
FD	0.13 (0.08)	0.13 (0.08)	0.15 (0.09)	0.371	0.691
**General cognition**
MMSE	28.97 (1.25)	27.98 (1.83)^**^	27.83 (1.80)^**^	5.655	0.005
**Memory**
IST	15.90 (3.17)	10.55 (3.41)^***^	9.38 (4.13)^***^	36.987	0.000
DST	14.92 (3.10)	8.81 (3.87)^***^	7.13 (4.38)^***^	45.504	0.000
AVLT	47.13 (11.59)	36.60 (11.20)^***^	33.88 (9.75)^***^	16.363	0.000
**Language**
SVF	21.28 (5.61)	18.45 (4.07)^*^	18.85 (4.74)^*^	4.01	0.021
**Attention**
TMT-A	34.21 (11.49)	36.33 (12.95)	38.20 (15.09)	0.896	0.411
**Executive**
TMT-B	90.28 (67.64)	104.07 (62.67)	105.80 (55.33)	0.743	0.478
**Visuospatial**
CDT	4.82 (0.39)	4.38 (1.04)^*^	4.48 (0.78)	3.428	0.036

*Data are mean ± standard deviations.*

* *represents p < 0.05,*

** *p < 0.01,*

*** *p < 0.001, respectively, meaning MCI-NA and MCI-A groups significantly different compared with NC. MMSE, Mini-Mental State Exam; AVLT, Auditory Verbal Learning Test; IST, Immediate Story Recall; DST, Delayed Story Recall; TMT-A, Trail-Making Test Part A; TMT-B, Trail-Making Test Part B; CDT, Clock-Drawing Test; SVF, Semantic Verbal Fluency; FD, framewise displacement*.

### Static and Dynamic ALFF Results

**Figure 2:**
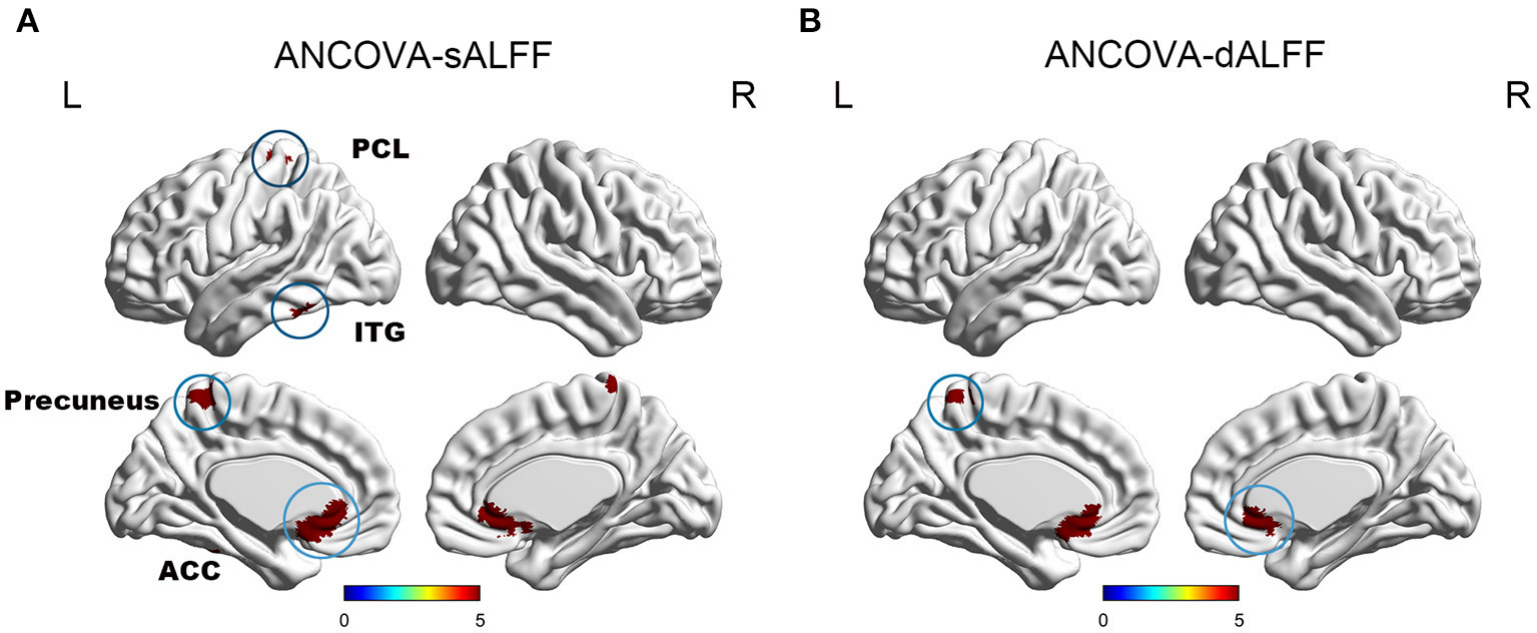
It illustrates the differences of sALFF and dALFF variance among NC, MCI-NA, and MCI-A. Specifically, **(A)** ANCOVA results of sALFF among NC, MCI-NA, and MCI-A (voxel *P* < 0.01, cluster *P* < 0.05, controlling for age, sex, education, and gray matter volume, GRF corrected); **(B)** ANCOVA results of dALFF variance among NC, MCI-NA, and MCI-A (voxel *P* < 0.01, cluster *P* < 0.05, controlling for age, sex, education, and gray matter volume, GRF corrected). sALFF, static amplitudes of low-frequency fluctuation; dALFF, dynamic amplitudes of low-frequency fluctuation; ACC, anterior cingulate cortex; ITG, inferior temporal gyrus; PCL, paracentral lobule.

**Figure 3:**
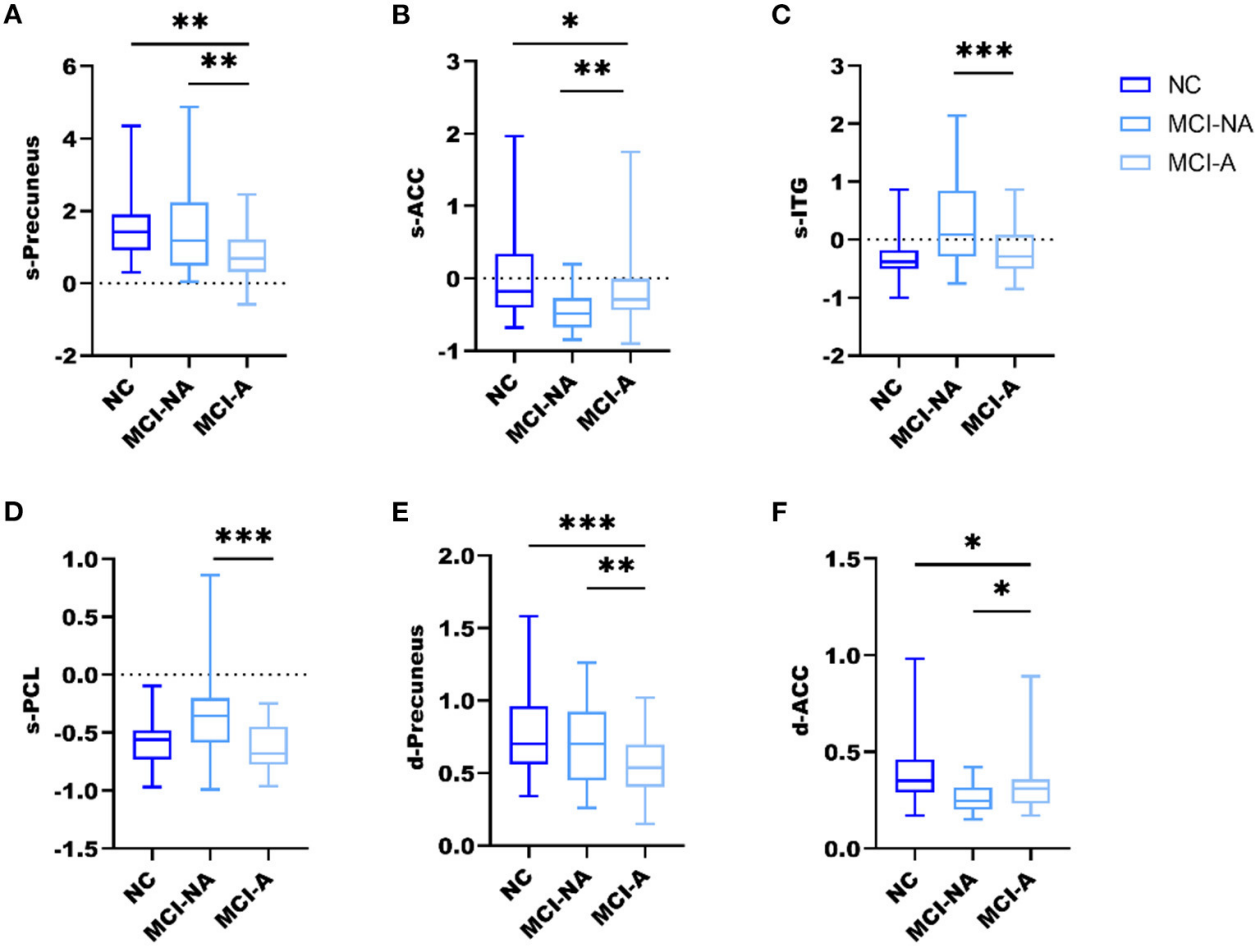
Box and whiskers illustrate the sALFF and dALFF variance differences among the three groups. Specifically, **(A)** the sALFF differences in the precuneus; **(B)** the sALFF differences in ACC; **(C)** the sALFF differences in ITG; **(D)** the sALFF differences in PCL; **(E)** the dALFF variance differences in the precuneus; **(F)** the dALFF variance differences in ACC; *, **, *** represents *p* < 0.05, *p* < 0.01, *p* < 0.001 after *post-hoc* analysis, meaning patient group MCI-A significantly different compared with MCI-NA and NC.

Mild cognitive impairment with anosognosia had increased sALFF in the ACC compared to MCI-NA and decreased sALFF compared to NC. In the precuneus, MCI-A showed decreased sALFF compared to both the MCI-NA and NC. In addition, MCI-A had decreased sALFF in the inferior temporal gyrus (ITG) and paracentral lobule (PCL) compared to MCI-NA. Regarding the dynamic metrics, MCI-A had increased dALFF variance in the ACC than MCI-NA, and decreased dALFF variance than NC. Decreased dALFF variance in the precuneus was observed in MCI-A compared to MCI-NA and NC (Figures 2, 3 and Table 2).

**Table 2:** Brain areas with significant sALFF and dALFF variance difference among three groups.

**Regions**	**Cluster**	**MNI Coordinate**			**Peak**
	**Voxels**	**X**	**Y**	**Z**	**Intensity**
**sALFF**
Left precuneus	22	−3	−54	69	9.17
Left ACC	30	−3	15	−12	8.55
Left ITG	18	−57	−48	−24	11.57
Left PCL	21	−12	−33	66	11.13
**dALFF variance**
Left precuneus	12	−6	−48	66	9.14
Right ACC	13	3	30	−3	8.91

*The difference of sALFF and dALFF variance between NC/MCI-NA/MCI-A groups (voxel P < 0.01, cluster P < 0.05, controlling for age, sex, education and gray matter volume, GRF corrected). sALFF, static amplitudes of low-frequency fluctuation; dALFF, dynamic amplitudes of low-frequency fluctuation; MNI, Montreal Neurological Institute; ACC, anterior cingulate cortex; ITG, inferior temporal gyrus; PCL, paracentral lobule*.

### Correlation Analysis

**Figure 4:**
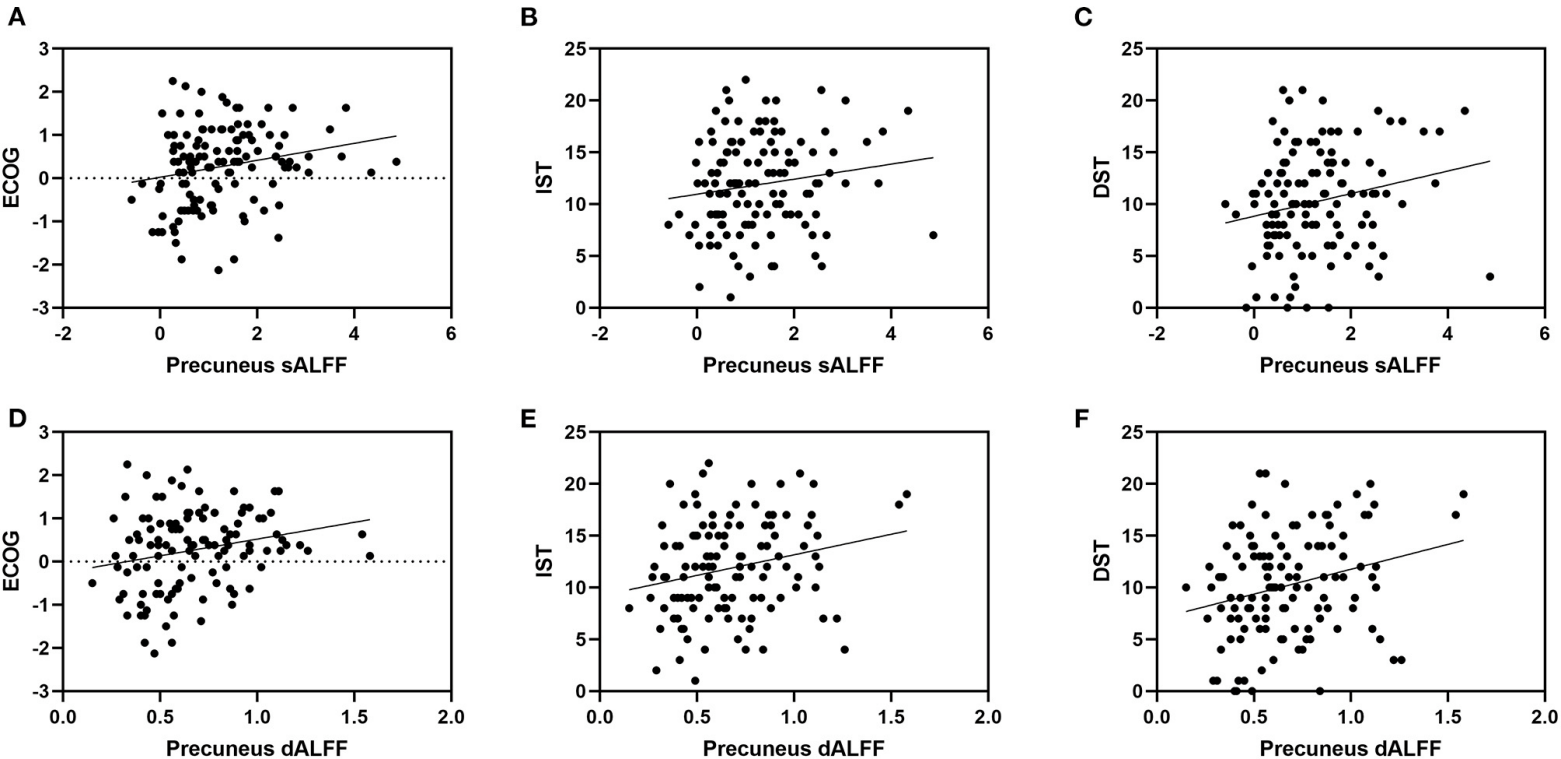
It shows the correlation between ALFF in precuneus and neuropsychological scores. Specifically, **(A)** represents the correlation between sALFF of precuneus and Ecog discrepancy score (*r*= 0.232, *p*= 0.012); **(B)** represents the correlation between sALFF of precuneus and IST scale (*r*= 0.200, *p*= 0.031); **(C)** represents the correlation between sALFF of precuneus and DST scale (*r*= 0.255, *p*= 0.006); **(D)** represents the correlation between dALFF variance of precuneus and Ecog discrepancy score (*r*= 0.235, *p*= 0.011); **(E)** represents the correlation between dALFF variance of precuneus and IST scale (*r*= 0.277, *p*= 0.003); **(F)** represents the correlation between dALFF variance of precuneus and DST scale (*r*= 0.298, *p*= 0.001). ECOG, Everyday Cognition questionnaire (discrepancy score; IST, Immediate Story Recall; DST, Delayed Story Recall.

We found no significant correlational relationship between the discrepancy score and MMSE score (*p* > 0.05, Supplementary Table 5). The sALFF and dALFF variance of the precuneus are significantly correlated with the Ecog discrepancy score (*r* = 0.232, *p* = 0.012; *r* = 0.235, *p* = 0.011). They are also correlated with IST (*r* = 0.200, *p* = 0.031; *r* = 0.277, *p* = 0.003) and DST scales (*r* = 0.255, *p* = 0.006; *r* = 0.298, *p* = 0.001). Correlation results showed that sALFF and dALFF variance of ACC are significantly related to IST (*r* = 0.355, *p* < 0.001; *r* = 0.408, *p* < 0.001) and DST scales (*r* = 0.322, *p* < 0.001; *r* = 0.352, *p* < 0.001). But we found no significant correlations between ALFF variance of ACC and Ecog discrepancy score (*p* > 0.05, Table 3 and Figure 4).

**Table 3:** Correlation between ALFF and neuropsychological scores among three groups corrected for age, sex, education, and gray matter volume.

	**s-Precuneus**		**d-Precuneus**		**s-ACC**		**d-ACC**	
	**Correlation** **coefficient**	***p*-value**	**Correlation** **coefficient**	***p*-value**	**Correlation ** **coefficient**	***p*-value**	**Correlation ** **coefficient**	***p*-value**
ECOG	0.232	0.012^b^	0.235	0.011^b^	−0.015	0.870	0.001	0.988
MMSE	0.102	0.274	0.080	0.393	0.034	0.714	0.121	0.196
IST	0.200	0.031^b^	0.277	0.003^a^	0.355	<0.001^a^	0.408	<0.001^a^
DST	0.255	0.006^b^	0.298	0.001^a^	0.322	<0.001^a^	0.352	<0.001^a^
AVLT	0.142	0.127	0.136	0.143	0.076	0.418	0.116	0.211
SVF	0.023	0.804	0.085	0.359	0.187	0.044^b^	0.209	0.024^b^
TMT-A	−0.019	0.839	0.064	0.496	−0.072	0.443	−0.092	0.323
TMT-B	−0.076	0.414	−0.099	0.289	−0.188	0.042	−0.141	0.129
CDT	−0.038	0.681	0.016	0.867	0.086	0.357	0.120	0.199

*ECOG, Everyday Cognition questionnaire (discrepancy score); MMSE, Mini-Mental State Exam; AVLT, Auditory Verbal Learning Test; IST, Immediate Story Recall; DST, Delayed Story Recall; TMT-A, Trail-Making Test Part A; TMT-B, Trail-Making Test Part B; CDT, Clock-Drawing Test; SVF, Semantic Verbal Fluency; s-Precuneus, Precuneus sALFF; d-Precuneus, Precuneus dALFF; s-ACC, Anterior cingulate cortex sALFF; d-ACC, Anterior cingulate cortex dALFF.*

a *p < 0.05, Bonferroni corrected.*

b *p < 0.05, uncorrected*.

## Discussion

For the first time, the current study explored the intrinsic brain activity change in MCI with anosognosia. The significant findings of the current study are as follows: (1) MCI-A had increased activity in the ACC and decreased activity in the precuneus, ITG, and PCL; (2) brain activity alternations in the precuneus were related to the memory function and anosognosia severity.

In the current study, MCI-A had decreased sALFF and dALFF variance in the precuneus relative to MCI-NA and NC. The default mode network (DMN) is a large-scale network consist of multiple brain regions including the PCC, precuneus, medial temporal lobes, and MPFC (Uddin et al., 2009; Therriault et al., 2018), and the precuneus is a functional core of the DMN (Utevsky et al., 2014). We found that the decreased sALFF and dALFF variance in the precuneus of MCI-A as previous studies reported that hypoactivation in DMN might be a sensitive and specific biomarker for incipient AD (Greicius et al., 2004; Rombouts et al., 2005). This suggested that anosognosia may be an early sign of AD in patients with MCI. On the contrary, the precuneus also plays a pivotal role in the CMS. The regions referred to CMS include the MPFC, ACC, PCC, and precuneus, and the function of CMS is associated with self-processing. Amongst, PCC and precuneus are related to the integration process, like the linkage between stimuli and personal context (Northoff and Bermpohl, 2004). A study suggested a central role for the precuneus in a broad spectrum of highly integrated tasks, including visuospatial imagery, episodic memory retrieval, and self-processing operations. Thus, the attenuated activation in the precuneus suggested that the damaged self-related or self-referential process may result in the anosognosia symptom. Decreased ALFF in the precuneus was also observed in patients with anosognosia in other diseases such as diffuse axonal injury (DAI) (Yao et al., 2015). Another study on schizophrenia with anosognosia also found reduced connectivity in the precuneus and ACC (Liemburg et al., 2012). In the previous studies, hypometabolism was found in MCI-A in the precuneus with the brain 18F-fluorodeoxyglucose PET (FDG-PET) (Nobili et al., 2010). Patients with MCI-A had significantly attenuated activation in CMS, indicating that a self-appraisal fMRI task is sensitive to functional brain changes associated with anosognosia in the patients with MCI (Ries et al., 2007). Thus, the precuneus is a critical region for understanding the mechanism behind anosognosia in patients with MCI. Nevertheless, whether the attenuated precuneus activation results from self-processing impairment or one of the early neuroimaging signs of the patients with MCI is still unknown, thus requires further studies to elaborate the neural mechanisms.

In this study, patients with MCI-A had increased activity in the ACC compared to the patients with MCI-NA. ACC is a part of a circuit involved in the formation of attention that regulates both cognitive and emotional processing, responsible for executive function (Margulies et al., 2007; Etkin et al., 2011). ACC might also be the hub in charge of error detection and error correction (Bush et al., 2000). Carter et al. observed a transient increase in the ACC activity using fMRI which occurred during incorrect responses to performances and provided evidence for the connection between ACC and error detection (Carter et al., 1998). ACC is not only a part of CMS but also an essential part of the self-referencing network (SRN). In a previous study, Bai et al. explored the changes of resting-state SRN in the MCI. They found that the dynamic changes are associated with increased functional connectivity at baseline and a more longitudinal diminish after follow-up (Bai et al., 2012). Another rs-fMRI study revealed that increased functional connectivity in the inferior frontal cortex and ACC is associated with anosognosia in schizophrenia spectrum disorders (Gerretsen et al., 2014). Amanzio et al. found that patients with AD with anosognosia showed reduced activation in the medial prefrontal circuit, particularly in the ACC during response inhibition (go/no-go) task, and proposed that ACC dysfunction of the executive monitoring system plays a particular role in anosognosia (Amanzio et al., 2011). Structural and hypometabolic alterations in ACC are also associated with anosognosia in patients with AD (Guerrier et al., 2018). Thus, we speculate that anosognosia is related to a temporary hyperactivation in ACC, maybe the reaction to the function of error detection of ACC. As the disease advances, hypoactivation could be seen in ACC. However, we need to further investigate the neuroimaging manifestations of anosognosia through long-term follow-up to validate our speculation.

After correcting for age, sex, education, and gray matter volume, we found that both sALFF and dALFF variance in the precuneus is positively related to the Ecog discrepancy score and recall delay scale. We, thus, hypothesized that abnormal activation in the precuneus is related to the clinical manifestations of patients with anosognosia, which showed poor performance on the memory tasks. This hypothesis is consistent with other studies revealing that the precuneus plays a significant role in memory (Wagner et al., 2005; Cavanna and Trimble, 2006).

Although no significant difference in the neuropsychological scores between MCI-A and MCI-NA was found, MCI-A had a tendency of decreased memory performance compared with MCI-NA. Previous research has confirmed that MCI-A performs worse on memory-related scales (Bregman et al., 2020). In this study, although no significant difference in memory performance between the MCI-A and MCI-NA was found, the activity strength of the precuneus brain area between the two groups has already been significantly different. Moreover, we found that the activity intensity of the precuneus is positively correlated with the memory scale score and anosognosia degree. These results implied that a significant decreased activity of the precuneus might account for and predict the memory function decline in patients with MCI-A compared to MCI-NA, which might provide an additional reference for the early clinical management of patients with MCI.

There is a limitation of the current study. There is still a lack of consensus in anosognosia diagnosis. There are three major ways to assess the lack of awareness (Clare, 2004; Vogel et al., 2004; Starkstein et al., 2006; Starkstein, 2014; Tondelli et al., 2018), which are as follows: (1) Clinician rating of awareness of the illness of the patients, which looks like a semi-structured interview dependent on the clinician judgment; (2) Prediction–performance discrepancies, the discrepancies are produced between expected performance and actual results of the patients; (3) patient-caregiver discrepancy scores. To better explore the anosognosia mechanism, a unified diagnostic standard is necessary.

## Conclusions

In conclusion, the abnormal brain activity in the precuneus and the ACC reflected by sALFF and dALFF supported the hypothesis that CMS regions play a vital role in the underlying neural mechanism behind anosognosia. Decreased activity in the precuneus may indicate a decline in memory function in patients with anosognosia.

## Data Availability Statement

The original contributions presented in the study are included in the article/Supplementary Material, further inquiries can be directed to the corresponding author/s.

## Ethics Statement

The studies involving human participants were reviewed and approved by the ethical standards of the institutional and/or national research committee and with the 1964 Helsinki declaration and its later amendments or comparable ethical standards. The patients/participants provided their written informed consent to participate in this study.

## Author Contributions

YF collected and analyzed the MRI data and wrote the first draft of the manuscript. XL analyzed the MRI data and wrote the protocol. QZ and KL designed and conceptualized the study and revised the manuscript. TZ, ZLi, LH, and XX assisted with study design and interpretation of findings. All the authors have contributed to reading and approving the final manuscript.

## Conflict of Interest

The authors declare that the research was conducted in the absence of any commercial or financial relationships that could be construed as a potential conflict of interest.

## Publisher's Note

All claims expressed in this article are solely those of the authors and do not necessarily represent those of their affiliated organizations, or those of the publisher, the editors and the reviewers. Any product that may be evaluated in this article, or claim that may be made by its manufacturer, is not guaranteed or endorsed by the publisher.
