# Supplementary material for: Effects of Anosognosia on Static and Dynamic Amplitudes of Low-Frequency Fluctuation in Mild Cognitive Impairment
Source: Front Aging Neurosci. 2021 Sep 7;13:705097. doi: 10.3389/fnagi.2021.705097 (PMC8867082; doi:10.3389/fnagi.2021.705097)

**Effects of Anosognosia on Static and Dynamic Amplitudes of Low Frequency Fluctuation in Mild Cognitive Impairment**

**Supplementary materials**

**Table S1** Brain areas with significant dALFF difference among three groups in other window sizes with 1TR as window step

|  | Region | Cluster | MNI Coordinate | | | Peak |
| --- | --- | --- | --- | --- | --- | --- |
|  |  | Voxels | X | Y | Z | Intensity |
| 14TR | Left ACC | 16 | -3 | 18 | -9 | 8.92 |
| 26TR | Left precuneus | 13 | -3 | -51 | 69 | 9.73 |
| 33TR | Left ITG | 15 | -57 | -48 | -24 | 13.2 |
|  | Left precuneus | 12 | -3 | -51 | 69 | 11.29 |

To test the reliability of the dALFF variance results, we also examined the results with window sizes of 14, 26, 33TR (1TR as window step) between NC/MCI-NA/MCI-A groups (voxel P<0.01, cluster P<0.05, controlling for age, sex, education and grey matter volume, GRF corrected). **Abbreviations:** ACC=anterior cingulate cortex; ITG=inferior temporal gyrus; PCL=paracentral lobule; MNI=Montreal Neurological Institute;

**Table S2** Brain areas with significant sALFF and dALFF variance (20TR window size) difference among three groups after correcting the head motion

|  | Regions | Cluster | MNI Coordinate | | | Peak |
| --- | --- | --- | --- | --- | --- | --- |
|  |  | Voxels | X | Y | Z | Intensity |
| sALFF |  |  |  |  |  |  |
|  | Left precuneus | 41 | -12 | -33 | 66 | 10.89 |
|  | Left ACC | 32 | -3 | 15 | -12 | 8.73 |
|  | Left ITG | 21 | -57 | -48 | -24 | 12.35 |
| dALFF variance | |  |  |  |  |  |
|  | Left precuneus | 12 | -6 | -48 | 66 | 8.82 |
|  | Right ACC | 12 | 3 | 30 | -3 | 8.97 |

The difference of sALFF and dALFF variance between NC/MCI-NA/MCI-A groups (voxel P<0.01, cluster P<0.05, controlling for age, sex, education, grey matter volume and head motion, GRF corrected). **Abbreviations:** sALFF=static amplitudes of low-frequency fluctuation; dALFF=dynamic amplitudes of low-frequency fluctuation; MNI=Montreal Neurological Institute; ACC=anterior cingulate cortex; ITG=inferior temporal gyrus;

**Table S3** Brain areas with significant dALFF difference among three groups in different window sizes with different window steps

| Window step | Window size | Region | Cluster | MNI Coordinate | | | Peak |
| --- | --- | --- | --- | --- | --- | --- | --- |
|  |  |  | Voxels | X | Y | Z | Intensity |
| 2TR | 14TR | Right ACC | 20 | 3 | 30 | -3 | 8.73 |
|  | 20TR | Left precuneus | 12 | -6 | -48 | 66 | 10.22 |
|  |  | Left ITG | 12 | -57 | -48 | -24 | 12.22 |
|  | 26TR | Left precuneus | 13 | -6 | -48 | 66 | 10.02 |
|  | 33TR | Left precuneus | 17 | -3 | -51 | 69 | 11.06 |
|  |  | Left ITG | 11 | -57 | -48 | -24 | 12.94 |
| 3TR | 14TR | Right ACC | 20 | 3 | 27 | -6 | 8.68 |
|  | 20TR | Left precuneus | 14 | -6 | -48 | 66 | 10.86 |
|  | 26TR | Left precuneus | 13 | -6 | -48 | 66 | 9.71 |
|  | 33TR | Left precuneus | 16 | -3 | -51 | 69 | 10.73 |
|  |  | Left ITG | 16 | -57 | -48 | -24 | 13.48 |

**Abbreviations:** dALFF=dynamic amplitudes of low-frequency fluctuation; MNI=Montreal Neurological Institute; ACC=anterior cingulate cortex; ITG=inferior temporal gyrus;

**Table S4** shows the subject IDs of ADNI

| ID | Research Group | ID | Research Group | ID | Research Group | ID | Research Group |
| --- | --- | --- | --- | --- | --- | --- | --- |
| 002_S_2043 | MCI | 014_S_4401 | CN | 037_S_4706 | MCI | 136_S_4408 | MCI |
| 002_S_2073 | MCI | 016_S_4952 | CN | 041_S_4427 | CN |  |  |
| 002_S_4171 | MCI | 018_S_2133 | MCI | 041_S_4510 | MCI |  |  |
| 002_S_4213 | CN | 018_S_2138 | MCI | 041_S_4874 | MCI |  |  |
| 002_S_4229 | MCI | 018_S_4313 | CN | 053_S_4578 | CN |  |  |
| 002_S_4237 | MCI | 018_S_4349 | CN | 053_S_4661 | MCI |  |  |
| 002_S_4262 | CN | 018_S_4400 | CN | 068_S_4067 | MCI |  |  |
| 002_S_4447 | MCI | 018_S_4597 | MCI | 068_S_4340 | CN |  |  |
| 002_S_4473 | MCI | 018_S_4809 | MCI | 068_S_4424 | CN |  |  |
| 002_S_4521 | MCI | 018_S_4868 | MCI | 068_S_4431 | MCI |  |  |
| 002_S_4654 | MCI | 018_S_4889 | MCI | 082_S_2121 | MCI |  |  |
| 002_S_4746 | MCI | 019_S_4285 | MCI | 082_S_4428 | CN |  |  |
| 002_S_4799 | MCI | 019_S_4293 | MCI | 094_S_4649 | CN |  |  |
| 005_S_4185 | MCI | 019_S_4367 | CN | 098_S_4003 | CN |  |  |
| 006_S_4346 | MCI | 019_S_4548 | MCI | 099_S_4076 | CN |  |  |
| 006_S_4357 | CN | 019_S_4680 | MCI | 100_S_2351 | MCI |  |  |
| 006_S_4363 | MCI | 021_S_4276 | CN | 100_S_4512 | MCI |  |  |
| 006_S_4449 | CN | 022_S_2379 | MCI | 100_S_4556 | MCI |  |  |
| 006_S_4485 | CN | 022_S_5004 | MCI | 116_S_4855 | CN |  |  |
| 006_S_4679 | MCI | 027_S_2219 | MCI | 127_S_2234 | MCI |  |  |
| 006_S_4713 | MCI | 027_S_4869 | MCI | 127_S_4197 | MCI |  |  |
| 006_S_4960 | MCI | 027_S_4919 | MCI | 127_S_4301 | MCI |  |  |
| 007_S_2394 | MCI | 029_S_2395 | MCI | 127_S_4604 | CN |  |  |
| 007_S_4516 | CN | 029_S_4384 | CN | 129_S_4220 | MCI |  |  |
| 007_S_4611 | MCI | 031_S_2018 | MCI | 129_S_4287 | MCI |  |  |
| 010_S_4345 | CN | 031_S_2022 | MCI | 129_S_4396 | CN |  |  |
| 010_S_4442 | CN | 031_S_4005 | MCI | 130_S_4294 | MCI |  |  |
| 011_S_4105 | CN | 031_S_4029 | MCI | 130_S_4405 | MCI |  |  |
| 011_S_4547 | MCI | 031_S_4032 | CN | 130_S_4415 | MCI |  |  |
| 012_S_4012 | MCI | 031_S_4042 | MCI | 130_S_4468 | MCI |  |  |
| 012_S_4026 | CN | 031_S_4149 | MCI | 130_S_4542 | MCI |  |  |
| 012_S_4128 | MCI | 031_S_4194 | MCI | 130_S_4605 | MCI |  |  |
| 012_S_4188 | MCI | 031_S_4218 | CN | 130_S_4817 | MCI |  |  |
| 013_S_4395 | MCI | 031_S_4476 | MCI | 130_S_4883 | MCI |  |  |
| 013_S_4579 | CN | 031_S_4721 | MCI | 130_S_4925 | MCI |  |  |
| 013_S_4580 | CN | 031_S_4947 | MCI | 135_S_4598 | CN |  |  |
| 013_S_4595 | MCI | 032_S_2119 | MCI | 135_S_4722 | MCI |  |  |
| 013_S_4616 | CN | 033_S_4176 | CN | 135_S_4723 | MCI |  |  |
| 013_S_4791 | MCI | 037_S_4028 | CN | 136_S_4189 | MCI |  |  |
| 013_S_4917 | MCI | 037_S_4308 | CN | 136_S_4269 | CN |  |  |

**Table S5** Correlation between the ECOG discrepancy score and neuropsychological scale

|  | ECOG discrepancy score | |
| --- | --- | --- |
|  | correlation  coefficient | p-value |
| MMSE | 0.099 | 0.286 |
| IST | 0.251 | 0.006 |
| DST | 0.293 | 0.001 |
| AVLT | 0.155 | 0.094 |
| SVF | -0.020 | 0.831 |
| TMT-A | -0.079 | 0.396 |
| TMT-B | -0.049 | 0.600 |
| CDT | 0.062 | 0.505 |

No significant correlation is found between the discrepancy score and MMSE score with the partial correlation after correcting age, sex and education (p>0.05). **Abbreviations:** ECOG= Everyday Cognition questionnaire; MMSE=Mini-Mental State Exam; AVLT=Auditory Verbal Learning Test; IST=Immediate Story Recall; DST=Delayed Story Recall; TMT-A=Trail-Making Test Part A; TMT-B=Trail-Making Test Part B; CDT=Clock-Drawing Test; SVF=Semantic Verbal Fluency;

**Table S6** The ECOG score in NC and MCI-NA groups

|  | NC | MCI-NA |  |
| --- | --- | --- | --- |
| n | 39 | 42 |  |
| ECOG-PT | 1.84(0.56) | 2.67(0.67) |  |
| ECOG-SP | 1.19(0.24) | 1.74(0.50) |  |
| ECOG discrepancy score | 0.64(0.46) | 0.93(0.57) |  |

**Abbreviations:** ECOG= Everyday Cognition questionnaire; PT= Participant Self-Report score; SP=Study Partner Report score; discrepancy score= PT-SP.

**Figure S1** ANCOVA results of dALFF variance in other window sizes with 1TR as window step


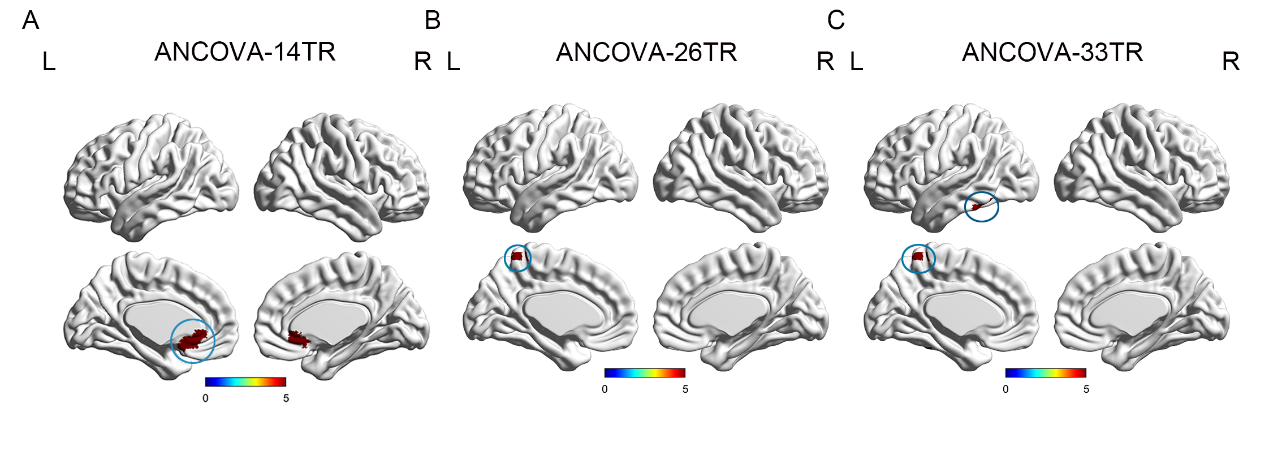


**A** **represents** ANCOVA results of dALFF variance among NC, MCI-NA and MCI-A in 14TR window size; **B represents** ANCOVA results of dALFF variance in 26TR window size; **C represents** ANCOVA results of dALFF variance in 33TR window size (voxel P<0.01, cluster P<0.05, controlling for age, sex, education and grey matter volume, GRF corrected).

**Figure S2** Box and whiskers illustrate the dALFF variance differences among three groups in other window sizes with 1TR as window step


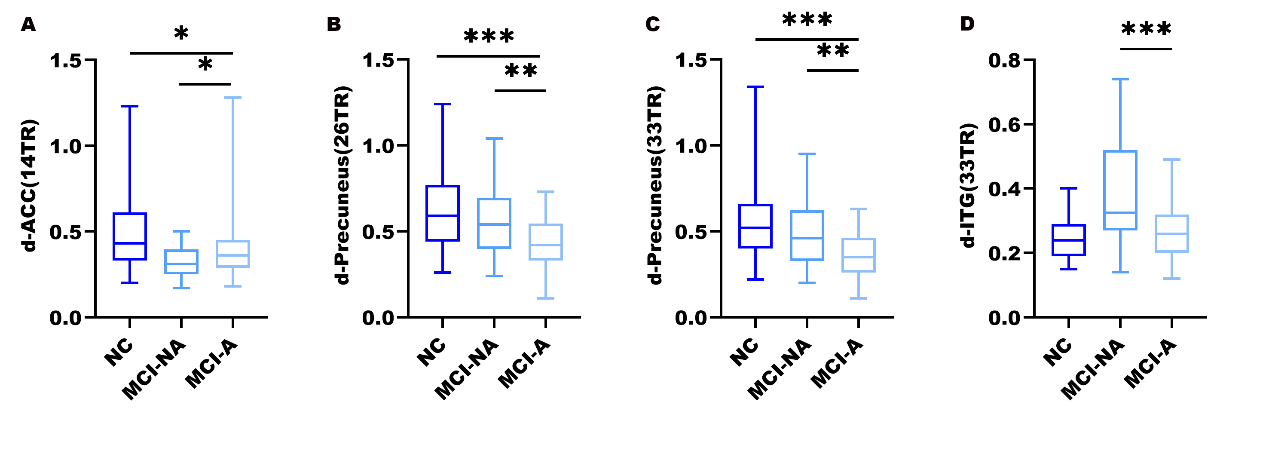


**A** **represents** the dALFF variance differences in ACC (14TR); **B represents** the dALFF variance differences in precuneus (26TR); **C represents** the dALFF variance differences in precuneus (33TR); **D represents** the dALFF variance differences in ITG (33TR); *, **, *** represents p < 0.05, p < 0.01, p < 0.001 after post-hoc analysis, meaning MCI-A significantly different compared with MCI-NA and NC.

**Figure S3** illustrates the dALFF variance differences among three groups in different window sizes with different window steps. Specifically, A, B, C, D represents differences in 14TR, 20TR, 26TR, 33TR window size with 2TR as window step. E, F, G, H represents differences in 14TR, 20TR, 26TR, 33TR window size with 3TR as window step.


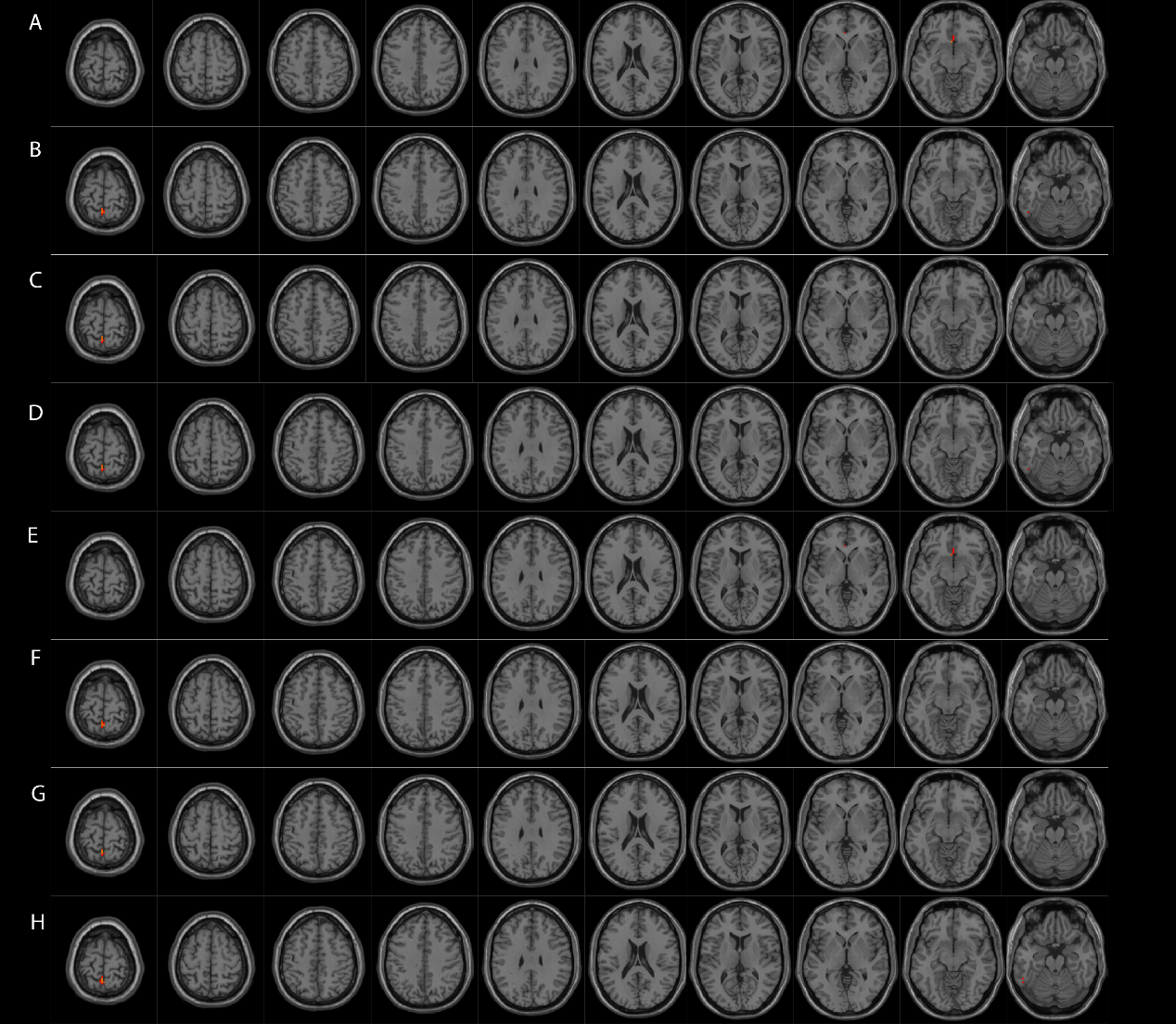

Supplement: Supplementary file 1 [file Data_Sheet_1.docx]
